# Comparison of outcomes for patients with and without a serious mental illness presenting to hospital for chronic obstruction pulmonary disease: retrospective observational study using administrative data

**DOI:** 10.1192/bjo.2023.522

**Published:** 2023-07-17

**Authors:** Sara Goldman, Anastasia Saoulidi, Sridevi Kalidindi, Eugenia Kravariti, Fiona Gaughran, Tim W. R. Briggs, William K. Gray

**Affiliations:** Institute of Psychiatry, Psychology and Neuroscience, King's College London, London, UK; Institute of Psychiatry, Psychology and Neuroscience, King's College London, London, UK; Getting It Right First Time Programme, NHS England, London, UK; and South London and Maudsley NHS Foundation Trust, London, UK; Institute of Psychiatry, Psychology and Neuroscience, King's College London, London, UK; and South London and Maudsley NHS Foundation Trust, London, UK; Getting It Right First Time Programme, NHS England, London, UK; and Department of Surgery, Royal National Orthopaedic Hospital, Stanmore, London, UK; Getting It Right First Time Programme, NHS England, London, UK

**Keywords:** Chronic obstructive pulmonary disease, mental illness, psychosis, schizophrenia, bipolar disorder

## Abstract

**Background:**

There are few data on the profile of those with serious mental illness (SMI) admitted to hospital for physical health reasons.

**Aims:**

To compare outcomes for patients with and without an SMI admitted to hospital in England where the primary reason for admission was chronic obstructive pulmonary disease (COPD).

**Method:**

This was a retrospective, observational analysis of the English Hospital Episodes Statistics data-set for the period from 1 April 2018 to 31 March 2019, for patients aged 18–74 years with COPD as the dominant reason for admission. Patient with an SMI (psychosis spectrum disorder, bipolar disorder) were identified.

**Results:**

Data were available for 54 578 patients, of whom 2096 (3.8%) had an SMI. Patients with an SMI were younger, more likely to be female and more likely to live in deprived areas than those without an SMI. The burden of comorbidity was similar between the two groups. After adjusting for covariates, SMI was associated with significantly greater risk of length of stay than the median (odds ratio 1.24, 95% CI 1.12–1.37, *P* ≤ 0.001) and with 30-day emergency readmission (odds ratio 1.51, 95% confidence interval 1.34–1.69, *P* ≤ 0.001) but not with in-hospital mortality.

**Conclusion:**

Clinicians should be aware of the potential for poorer outcomes in patients with an SMI even when the SMI is not the primary reason for admission. Collaborative working across mental and physical healthcare provision may facilitate improved outcomes for people with SMI.

Severe mental illnesses (SMIs) are a cluster of mental disorders including schizophrenia, schizoaffective disorder and bipolar disorder that can result in substantial deterioration of social and occupational functioning, long-term disability and reduction in quality of life.^1^ In the UK, the prevalence of SMI is estimated to be around 0.9%.^2^ Apart from the psychosocial effects, premature mortality appears to occur more often in people with SMI compared with those with other mental disorders and/or the general population, with a reduction in life expectancy of as much as 10 years.^1^ A large proportion of the observed premature deaths can be accounted for by physical health problems. Evidence suggests that as many as 61.8% of all SMI deaths occur because of circulatory and respiratory disorders and cancers.^3^

A report by the Nuffield Trust looked at the differences in hospital use in people aged less than 75 years with mental health difficulties (SMI and non-SMI) and physical health problems.^4^ They found that people with mental health difficulties were almost four times more likely to use emergency care services than other patient groups, with the most care needed for physical health problems. Among all hospital patients with mental health difficulties, those with SMI had higher rates of emergency in-patient admissions and out-patient care. Furthermore, levels of socioeconomic deprivation were highly associated with emergency care use, and patients with SMI had higher levels of deprivation compared with those with other mental health difficulties and physical health problems. Hospital patients with mental health difficulties were also more likely to stay in hospital for a longer period. There is evidence that people with SMI are more likely than the general population to have physical multimorbidity (more than one comorbid physical condition), which can also contribute to adverse health outcomes and affect hospital use.^5^

Although there is a good evidence base for adverse health and hospital use outcomes in people with SMI, there has been much less work to assess the interactions between various factors associated with outcomes. Furthermore, there appears to be a gap in research regarding the differences among patients within different SMIs. In the general population, evidence suggests that the overall relative mortality risk is higher for people with schizophrenia compared with people with bipolar disorder.^6,7^ Within hospital populations, a study of 43 817 Medicaid enrolees in the USA found that people with schizophrenia had longer hospital stays, a greater number of physical comorbidities and higher 30-day hospital readmission rates compared with people with bipolar and depressive disorders.^8^ However, data from the USA may not readily translate to countries with different healthcare funding models. Greater understanding of the factors associated with discrepancies in health outcomes for people with SMIs can inform the development of strategies to address them.

The aim of the current study was to analyse an administrative data-set for England to explore factors associated with poor outcomes from hospital admission for younger adult patients (18–74 years old) with a specific physical health condition, chronic obstructive pulmonary disease (COPD), focusing on the presence of SMI and subgroups of SMIs. Our interest was in hospital admissions for reasons other than an SMI but where the SMI could be a complicating factor in symptom management. COPD was chosen as it is a common physical health condition with relatively high rates of hospital admission owing to exacerbation of symptoms. People with SMI have been shown to have high rates of respiratory disease, and that respiratory disease is associated with premature mortality in people with SMI.^9^ People with schizophrenia have higher rates of COPD than the general population and are more likely to die of infections such as pneumonia and influenza, common precipitants for COPD.^10^ Our primary hypothesis was that people admitted to hospital with COPD who also had a SMI would have poorer outcomes than those admitted with COPD who did not have a SMI. The findings of the study should help clinicians and service managers to better understand the needs of people with a SMI when they are admitted to hospital, even if the primary reason for the admission was not the SMI.

## Method

### Ethics

Data analysis and presentation conformed to the current guidelines recommended by the National Health Service (NHS) in relation to using Hospital Episodes Statistics (HES) data-sets for care quality improvement purposes.^11,12^ The data were anonymised to the required level recommended by the ISB1523 Anonymisation Standard for Publishing Health and Social Care Data.^13^ Formal ethical approval for the study was not required because it did not directly involve human participants. This study was completed in accordance with the Helsinki Declaration as revised in 2013. Informed consent was not sought for the present study because it was an analysis of routine clinical data and did not directly involve human participants.

### Study design and data collection

This was an exploratory, retrospective cohort study using administrative hospital activity data. Patients were followed up post-discharge through data linkage to subsequent hospital stays at a patient level. NHS Digital collects HES data for all patients admitted to hospitals in England where patient care is funded by the NHS. This includes patients admitted to private hospitals where their care was funded by the NHS. The data are collected predominantly to allow NHS trusts (which govern secondary and tertiary care hospitals in England) to be funded for providing hospital care. Data collection is mandatory, and data are entered by trained clinical coders from each trust.

### Timing, case ascertainment, inclusion and exclusion criteria

HES data were extracted for the period from 1 April 2018 to 31 March 2019 for patients aged 18–74 years with COPD as the dominant diagnosis recorded as the disorder leading to the hospital stay. The discharge date, rather than the admission date, defined the data collection period, and patients who died during their stay were included. The study period was chosen as it was the last complete financial year of data prior to the COVID-19 pandemic, and this defined the sample size. COPD was identified using the ICD-10 codes J40–J44 inclusive. The descriptions associated with these codes are given in Supplementary Table 1 available at https://doi.org/10.1192/bjo.2023.522. Patients aged ≥75 years were excluded as it was felt that issues related to age-related decline would otherwise dominate the data-set. This strategy is consistent with that of an earlier Nuffield Trust study on this topic.^4^ In instances where a patient underwent repeated hospital admissions over the course of the study period, only the first admission was retained. This ensured that all data points were independent at a patient level and avoided biasing the data with repeat admissions.

### Exposure

The primary exposure of interest was an SMI diagnosis recorded during the admission. Patients were identified as having an SMI if they had any of the following ICD-10 codes recorded in any position in their diagnostic record during the hospital stay: F20–29 (psychosis spectrum disorder), F30–31 (bipolar disorder). The descriptions associated with these codes are given in Supplementary Table 1.

In the sub-analysis, those with a non-affective psychosis spectrum disorder and those with a bipolar disorder were compared. Individuals who did not have an SMI or who had diagnoses of a psychosis spectrum disorder and a bipolar disorder were excluded from this sub-analysis (see results section for number of patients).

### Outcomes

For this exploratory analysis, three outcome measures, where data were available, were deemed relevant.

- Length of stay greater than the median (2 days). Length of stay data were dichotomised as the data were non-normally distributed with a long tail of long stays but little variability for the majority of patients, meaning that it was not possible to normalise the distribution.
- Emergency readmission within 30 days of discharge. This was only recorded where the patient stayed for 1 night or more in hospital and was recorded for any cause. The occurrence of the event was recorded if one or more readmissions occurred.
- In-hospital mortality. Mortality data were available from the UK Office for National Statistics and were recorded for any cause.

The values report for readmission and mortality can be thought of in terms of cumulative incidence. Although all three outcomes were chosen *a priori* and are NHS England standard outcome metrics, the exact method of analysis for length of stay was decided *post hoc* based on the data distribution.

### Covariates

- Age: categorised into the following age groups: 18–49 years, 50–54 years, 55–59 years, 60–64 years, 65–69 years, and 70–74 years. Categorisation of the data was felt to make it more meaningful and easier to interpret for this exploratory analysis.
- Sex: male or female.
- Deprivation: recorded using the Index of Multiple Deprivation for the lower-layer super output area (LSOA) of the patient's home address, with scores categorised into quintiles based on national averages.^14^ England is divided into 32 844 geographically defined LSOAs by the Office for National Statistics, each with typically 1400–2000 residents.
- Comorbidities: 17 comorbidities were taken from those used to construct the Charlson Comorbidity Index.^15^ The index uses ICD-10 codes to identify the following comorbidities: peripheral vascular disease, chronic heart failure, acute myocardial infarction, cerebrovascular disease, dementia, pulmonary disease, connective tissue disease, peptic ulcer, diabetes (with and without chronic complications), hemiplegia/paraplegia, renal disease, primary cancer, metastatic cancer, mild liver disease, severe liver disease and HIV/AIDS. A comorbidity was deemed present if it was recorded in HES as a secondary diagnosis in the index admission or as a primary or secondary diagnosis in any admission during the previous year.^15^
- Elective or emergency admission. Elective admissions are planned admissions, often for a surgical procedure, and include day-case surgery.^16^

### Data management and statistical analyses

Standard statistical software was used to conduct the analysis: Microsoft Excel (Microsoft Corp.), Stata (StataCorp LLC) and IBM SPSS (version 27, IBM Corp.). In the descriptive analysis, data were categorised as detailed above and summarised in terms of frequency and percentage. Length of stay was summarised using the median and interquartile range. Relationships between presence of an SMI and outcomes were explored with a multilevel, multivariable logistic regression model, using the melogit command in Stata. A two-level (patients nested within NHS hospital trusts) intercept-only model was constructed. NHS hospital trust was entered as a random effect to allow for clustering of cases within trusts. All models were adjusted for the covariates age (categorised into six age bands as described above), sex, deprivation quintile, the 17 comorbidities listed above (each entered as separate covariate as dichotomous variables (disease present versus disease absent)) and emergency versus elective admission.

Only in the case of deprivation were there missing values. These were for patients who did not have a permanent residence in England. Given the small numbers involved, no attempt was made to impute missing values. The models were summarised in terms of odds ratios and 95% confidence intervals. Confidence intervals were calculated based on the observed information matrix (inverse of the negative Hessian matrix) for the log-likelihood. Statistical significance was indicated by an odds ratio of a 95% confidence interval not crossing 1.

## Results

The data extraction process yielded a data-set of 54 578 patients, of whom 2096 (3.8%) had an SMI. Table 1 summarises the demographic, socioeconomic and comorbidity profiles of the cohort according to whether they had or did not have an SMI. Compared with those without an SMI, those with an SMI had a noticeably younger age profile and were slightly more likely to be female and from a more deprived area. In both the SMI and non-SMI groups, almost all admissions were non-elective. Unsurprisingly, pulmonary disease was the most common comorbidity. Although the SMI group had a younger age profile, many age-related comorbidities were more common in this group than in the non-SMI group. These included pulmonary disease, diabetes, chronic heart failure, liver disease, renal disease and dementia.

**Table 1:** Demographic and comorbidity profiles and outcomes of COPD patients with and without an SMI

Variable	Without SMI (*n* = 52 482)	With SMI (*n* = 2096)
Age band (years)		
18–49	4333 (8.3%)	287 (13.7%)
50–54	4053 (7.7%)	306 (14.6%)
55–59	6220 (11.9%)	353 (16.8%)
60–64	8928 (17.0%)	399 (19.0%)
65–69	12 333 (23.5%)	410 (19.6%)
70–74	16 615 (31.7%)	341 (16.3%)
Sex		
Female	27 484 (52.4%)	1144 (54.6%)
Male	24 998 (47.6%)	952 (45.4%)
Deprivation quintile^a^ (missing = 1705)		
1 (most deprived)	18 689 (35.6%)	831 (39.6%)
2	12 021 (22.9%)	509 (24.3%)
3	9027 (17.2%)	327 (15.6%)
4	6676 (12.7%)	210 (10.0%)
5 (least deprived)	4477 (8.5%)	106 (5.1%)
Charlson Comorbidity Index^b^		
Peripheral vascular disease	2493 (4.8%)	45 (2.1%)
Chronic heart failure	5228 (10.0%)	192 (9.2%)
Acute myocardial infarction	5015 (9.6%)	142 (6.8%)
Cerebrovascular disease	1313 (2.5%)	66 (3.1%)
Dementia	817 (1.6%)	53 (2.5%)
Pulmonary disease	31 170 (59.4%)	1323 (63.1%)
Connective tissue disease	2120 (4.0%)	44 (2.1%)
Peptic ulcer	259 (0.5%)	10 (0.5%)
Diabetes without chronic complications	9048 (17.2%)	509 (24.3%)
Diabetes with chronic complications	448 (0.9%)	34 (1.6%)
Hemiplegia/paraplegia	474 (0.9%)	20 (1.0%)
Renal disease	2913 (5.6%)	145 (6.9%)
Primary cancer	3100 (5.9%)	58 (2.8%)
Metastatic carcinoma	979 (1.9%)	23 (1.1%)
Mild liver disease	2172 (4.1%)	158 (7.5%)
Severe liver disease	198 (0.4%)	5 (0.2%)
HIV/AIDS	116 (0.2%)	3 (0.1%)
Elective admission	2978 (5.7%)	42 (2.0%)
Median length of stay (days)	2 (IQR 1–5)	3 (IQR 1–6)
Length of stay greater than median (2 days)	23 862 (45.5%)	1056 (50.4%)
30-day emergency readmission	7581 (14.4%)	401 (19.1%)
Mortality during hospital admission	1014 (1.9%)	40 (1.9%)

SMI, serious mental illness; IQR, interquartile range.

a. Deprivation quintile is based on the Index of Multiple Deprivation score.

b. For the Charlson Comorbidity Index items, only those with the disease are listed. Individual patients can appear in multiple disease categories.

Patient outcomes are also summarised in Table 1. Median stay was longer for patients with SMI than those without. Despite the younger age structure of the SMI group, they had a higher rate of 30-day emergency readmissions and a similar mortality rate to the non-SMI group.

The exploratory multilevel, multivariable logistic regression model outputs are summarised in Table 2. SMI was associated with significantly greater odds of a length of stay greater than the median and 30-day emergency readmission but not in-hospital mortality.

**Table 2:** Summary of multivariable multilevel logistic regression models^a^ for factors associated with SMI

	Length of stay greater than median	30-day emergency readmission	In-hospital mortality
Variable	Odds ratio (95% CI)	Odds ratio (95% CI)	Odds ratio (95% CI)
People with a diagnosis of SMI compared with those without an SMI diagnosis	1.24 (1.12–1.37), *P* ≤ 0.001	1.51 (1.34–1.69), *P* ≤ 0.001	1.26 (0.90–1.74), *P* = 0.173
People with a diagnosis of bipolar disorder compared with those with a non-affective psychosis	1.02 (0.81–1.30), *P* = 0.840	1.17 (0.90–1.50), *P* = 0.236	0.39 (0.16–0.95), *P* = 0.038

SMI, serious mental illness.

a All models were adjusted for age, sex, deprivation, comorbidities and elective versus emergency admission.

A sub-analysis compared those with non-affective psychosis spectrum disorders only (*n* = 1188, 2.2%) and bipolar disorders only (*n* = 712, 1.3%). Both diagnoses were recorded in 196 (0.4%) patients, and these patients were excluded. Those with bipolar disorders were more likely to be female, although the groups were similar across other demographic variables and in terms of their comorbidity profile (Table 3). Multilevel, multivariable logistic regression model outputs are shown in Table 2. The odds of in-hospital mortality in those with bipolar disorder were significantly lower than in those with non-affective psychosis spectrum disorders. There was no difference in the odds of 30-day emergency readmissions or length of stay greater than the median.

**Table 3:** Demographic and comorbidity profile and outcomes of COPD patients with non-affective psychosis spectrum disorder and bipolar disorder

Variable	Non-affective psychosis spectrum disorder (*n* = 1188; 62.5%)	Bipolar disorder (*n* = 712; 37.5%)
Age band (years)		
18–49	160 (13.5%)	92 (12.9%)
50–54	173 (14.6%)	102 (14.3%)
55–59	203 (17.1%)	123 (17.3%)
60–64	227 (19.1%)	135 (19.0%)
65–69	232 (19.5%)	145 (20.4%)
70–74	193 (16.2%)	115 (16.2%)
Sex		
Female	504 (42.4%)	514 (72.2%)
Male	684 (58.6%)	198 (28.8%)
Deprivation quintile^a^ (missing = 104)		
1 (most deprived)	470 (39.6%)	274 (38.5%)
2	288 (24.2%)	179 (25.1%)
3	190 (16.0%)	102 (14.3%)
4	111 (9.3%)	85 (11.9%)
5 (least deprived)	55 (4.6%)	42 (5.9%)
Number of Charlson Index comorbidities recorded^b^		
0	227 (19.1%)	138 (19.4%)
1	526 (44.3%)	317 (44.5%)
2	291 (24.5%)	161 (22.6%)
3	106 (8.9%)	67 (9.4%)
≥4	38 (3.2%)	29 (4.1%)
Elective admission	23 (1.9%)	15 (2.1%)
Median length of stay	3 (IQR 1–6)	2 (IQR 1–6)
Length of stay > the median (2 days)	599 (50.4%)	355 (49.9%)
30-day emergency readmission	211 (17.8%)	150 (21.1%)
Mortality during hospital admission	29 (2.4%)	7 (1.0%)

IQR, interquartile range.

a. Deprivation quintile is based on the Index of Multiple Deprivation score.

b. Charlson Index comorbidities are those recorded as a secondary diagnosis in the index admission or as a primary or secondary diagnosis in any admission during the previous year.

## Discussion

Our study explored the associations between the presence or absence of a SMI and outcomes for people aged 18–74 years who were admitted to hospital, where the primary reason for admission was not the SMI but a physical health condition. Focusing on a specific physical health condition, COPD, meant that our study cohort was relatively homogenous. We were also able to adjust for the presence of potential confounders. The presence of an SMI was associated with longer hospital stay and higher odds of early emergency readmissions. In the sub-analysis, patients with non-affective psychosis had higher odds of mortality than those with bipolar disorder.

Our work builds on the 2015 Nuffield Trust study by exploring in detail a specific physical health condition.^4^ There is limited published material regarding outcomes for people with an SMI who are admitted to hospital, and most such studies have been small scale and/or single site.^17,18^ However, some larger-scale studies have been published. A retrospective analysis of administrative data from 16 898 patients admitted to hospital in Tasmania, Australia, was published in 2018.^19^ The authors investigated length of stay for patients admitted with five chronic health conditions (lung or colorectal cancer, COPD, type II diabetes, ischaemic heart disease and stroke) and identified a significantly longer hospital stay for patients with a mental illness compared with those without. A Canadian study of 12 283 patients aged 65 years and older admitted to an acute care hospital in Newfoundland noted longer stay, higher readmission rates and greater cost of care for patients with mental illness.^20^ Overall, 8.3% of the older adults admitted to hospital had a mental illness. The Nuffield Trust report identified that SMI patients experienced 6.7 times more emergency admissions and greater length of stay than patients without an SMI.^4^ A study using the US Nationwide Readmissions Database for 6 328 613 medical and surgical admissions reported a 30-day medical readmission rate of 23.1% for patients with SMI versus 13.8% for patients without a SMI (adjusted odds ratios, 1.80, 95% CI 1.77–1.83).^21^ Our study broadly supports the findings of all these previous studies despite methodological differences and differences in setting.

Our study did not seek to explore possible causes of poorer outcomes for people with an SMI who are admitted to hospital. However, previous research has suggested that impaired patient cognition, lack of involvement in patient self-care and rehabilitation, and perceived social stigma around raising SMI as a potential factor in hospital care could all contribute.^22^ Healthcare systems often require patients to actively seek help and support. This might pose a challenge for individuals with a diagnosis of SMI for a variety of reasons. Certain symptom profiles, such as difficulties with executive functioning, lack of knowledge, stigma and reduced social support can affect people's awareness, insight, motivation and ability to explain physical experiences.^5,23^ This can prevent help-seeking. Once help has been sought, although healthcare professionals report good understanding of the importance of addressing physical health needs in people with SMI, lack of training, uncertainty and limited resources have been identified as barriers to care provision.^24^ Further investigation of the mechanisms by which an SMI influences outcomes of hospital admissions is needed if outcomes are to be improved.

We report a significantly higher mortality rate in patients with non-affective psychosis compared with those with bipolar disorder. Overall, the increased mortality rate and risk in schizophrenia found in the current study is consistent with the existing literature. One comprehensive study of all people diagnosed with schizophrenia or bipolar disorder in Denmark from 1965 to 2014 found that the standardised mortality ratio for schizophrenia was two times higher than that for bipolar disorder.^25^ A systematic review and meta-analysis of factors associated with mortality in schizophrenia found that whereas there were no differences in risk between schizophrenia and other psychiatric disorders overall, there was a difference in risk ratios between schizophrenia and bipolar disorder specifically, with people with schizophrenia being at a higher risk of mortality.^6^

We were unable to comment on the effect of medication use, as such data are not collected in HES. Although use of second-generation antipsychotics has been linked to increased risk of pneumonia in people with SMI,^26^ all-cause mortality is higher in people with schizophrenia who are not prescribed anti-psychotic medication, and long-term benefits of treatment adherence in schizophrenia appear to counter the load created by adverse effects of medication.^26,27^ One observational study showed that compared with people with bipolar disorder, people with schizophrenia had lower insight; this contributed to poorer medication adherence and worse therapeutic alliance with healthcare professionals, which may adversely affect physical health outcomes.^28^ Although medication may affect mortality rates and other adverse health outcomes in schizophrenia, its effects are likely to be dependent on a variety of factors, including attitudes towards medication and insight, dosing load, polypharmacy and potential medication interactions, as well as person-specific vulnerability factors. People with schizophrenia are more likely to have a poor diet, smoke more and exercise less, irrespective of socioeconomic status and medication use; this may also affect outcomes.^29^

The SMI cohort was younger than the non-SMI cohort. Younger age may reflect the additional health burden of SMI, with COPD becoming more of a health burden at a younger age. The SMI cohort were also more likely to be female, although most of this excess was due to a large predominance of females in the subgroup of patients with bipolar disorder. Affective SMIs are known to be more common in females.^30^

### Strengths and limitations

This study has many strengths. It is the largest study of its kind to date. Furthermore, the study augments the paucity of data on length of stay for patients with COPD and an SMI. It is also the first study to investigate hospital usage in COPD patients with SMI compared with the reference population. We analysed data for the entire hospital-admitted population of a national healthcare system and were able to link outcomes over time and over different healthcare providers; thus, the risk of bias in the patient population when considering hospital-admitted populations should be low, at least within England and other countries with similar healthcare funding models. Further, our results may have relevance beyond acute hospital care, although we would caution against interpreting them as directly relevant to other settings. Studies examining the outcomes of patients with SMI in the ambulatory and community setting are warranted.

We chose to exclude patients aged <18 years and ≥75 years from our cohort. For older patients, it was felt that age-related health problems would be the dominant influence on outcomes and so mask associations in younger patients. Although findings would probably be quite different if the study were repeated in patients aged ≥75 years, we do not suggest that older patients with COPD and a SMI would not have poorer outcomes than those with COPD without an SMI, just that any association may be harder to identify. A similar study in older age groups and for people with different physical health conditions is merited.

Several limitations must also be acknowledged. As this was a retrospective study, the data were collected for administrative purposes and not primarily for research. As such, some SMIs and comorbidities may not have been recorded in the diagnostic record. Although validation of large administrative data-sets is difficult owing to the lack of a criterion standard against which to assess accuracy, members of our team have recently published a study investigating the consistency of coding diagnoses that are mandatory in HES.^31,32^ Levels of consistency of coding of diagnoses from the first to a subsequent hospital stay varied from 91.4% for diabetes to 56.3% for autism, suggesting that this is an important issue for some conditions.

The administrative nature of the data meant there was a limited amount of information regarding presentation. Therefore, we were not able to quantify the severity of presenting COPD symptoms in each patient, nor the seriousness of each patient's mental health symptoms. Furthermore, the study only considered hospital usage and thus included very little information about care beyond the acute sector. There is a lack of data on some patient characteristics such as smoking status and health-related behaviours. Tobacco use is not consistently recorded in HES, and so we chose not to include it as a covariate in modelling. Current smoking status may well confound our findings, for example, if current smoking was more common in patients with SMI.^33,34^ However, as the vast majority of our cohort will have been moderate or heavy tobacco smokers (even if not currently), this is unlikely to fully explain our findings. Given evidence that smoking cessation interventions can be successful in people with SMI,^35,36^ the link between smoking and outcomes for people with SMI who are admitted to hospital should be further investigated.

### Implications

Patients with COPD and a comorbidity of SMI had poorer outcomes than those with COPD and no SMI. Patients with a diagnosis of schizophrenia had higher mortality rates when admitted for their COPD than those with a diagnosis of bipolar disorder. Understanding more clearly the reasons for this will be key to improving patient outcomes. Clinicians need to be aware that people with an SMI are at particular risk of poor outcomes when admitted to hospital and should seek specialist advice to support medication adherence, make suitable adjustments to care provision during a hospital stay and ensure effective discharge planning.

As we move to integrated care boards across England, there is an opportunity to provide more holistic and joined-up physical healthcare for people with SMI, with the aims over time of taking a more preventive approach, reducing the premature mortality gap and supporting improved use of healthcare resources.

## Data Availability

Requests for any underlying data used in our analysis cannot be granted by the authors as the data are calculated from data under licence from NHS Digital, where conditions of use (and further use) apply. Therefore, we are unable to make our data available in a repository. However, HES data are available from NHS Digital upon request. The analytic code supporting the findings has not been made available to other researchers within a repository as it is relatively trivial. Sufficient details are presented in the Method section to allow reproduction of our analysis.

## Supplementary material

For supplementary material accompanying this paper visit http://doi.org/10.1192/bjo.2023.522. click here to view supplementary material

## References

[1] Walker ER, McGee RE, Druss BG. Mortality in mental disorders and global disease burden implications: a systematic review and meta-analysis. JAMA Psychiatry 2015; 72(4): 334–41. 25671328 10.1001/jamapsychiatry.2014.2502 PMC4461039

[2] Public Health England. Severe Mental Illness (SMI) and Physical Health Inequalities. UK Government, 2018 (https://www.gov.uk/government/publications/severe-mental-illness-smi-physical-health-inequalities/severe-mental-illness-and-physical-health-inequalities-briefing).

[3] John A, McGregor J, Jones I, Lee SC, Walters JTR, Owen MJ, et al. Premature mortality among people with severe mental illness – new evidence from linked primary care data. Schizophr Res 2018; 199: 154–62. 29728293 10.1016/j.schres.2018.04.009

[4] Dorning H, Davies A, Blunt I. Focus On: People with Mental Ill Health and Hospital Use. Nuffield Trust, 2015.

[5] Filipcic IS, Bajic Z, Filipcic I. The onset and accumulation of physical multimorbidity in severe and common mental disorders. Curr Opin Psychiatry 2020; 33(5): 484–90. 32639364 10.1097/YCO.0000000000000635

[6] Correll CU, Solmi M, Croatto G, Schneider LK, Rohani-Montez SC, Fairley L, et al. Mortality in people with schizophrenia: a systematic review and meta-analysis of relative risk and aggravating or attenuating factors. World Psychiatry 2022; 21(2): 248–71. 35524619 10.1002/wps.20994 PMC9077617

[7] Laursen TM. Life expectancy among persons with schizophrenia or bipolar affective disorder. Schizophr Res 2011; 131(1-3): 101–4. 21741216 10.1016/j.schres.2011.06.008

[8] Cook JA, Burke-Miller JK, Jonikas JA, Aranda F, Santos A. Factors associated with 30-day readmissions following medical hospitalizations among Medicaid beneficiaries with schizophrenia, bipolar disorder, and major depressive disorder. Psychiatry Res 2020; 291: 113168. 32619823 10.1016/j.psychres.2020.113168

[9] Suetani S, Honarparvar F, Siskind D, Hindley G, Veronese N, Vancampfort D, et al. Increased rates of respiratory disease in schizophrenia: a systematic review and meta-analysis including 619,214 individuals with schizophrenia and 52,159,551 controls. Schizophr Res 2021; 237: 131–40. 34521040 10.1016/j.schres.2021.08.022

[10] Olfson M, Gerhard T, Huang C, Crystal S, Stroup TS. Premature mortality among adults with schizophrenia in the United States. JAMA Psychiatry 2015; 72(12): 1172–81. 26509694 10.1001/jamapsychiatry.2015.1737

[11] NHS Digital. Hospital Episode Statistics (HES) Analysis Guide. NHS Digital, 2018.

[12] NHS Digital. Users, Uses and Access to Hospital Episode Statistics. NHS Digital, 2019 (https://digital.nhs.uk/data-and-information/data-tools-and-services/data-services/hospital-episode-statistics/users-uses-and-access-to-hospital-episode-statistics).

[13] Information Standard Board for Health and Social Care. Anonymisation Standard for Publishing Health and Social Care Data Specification (Process Standard). NHS Digital, 2013.

[14] Office for National Statistics. Lower Layer Super Output Area Population Estimates. Office for National Statistics, 2020 (https://www.ons.gov.uk/peoplepopulationandcommunity/populationandmigration/populationestimates/datasets/lowersuperoutputareamidyearpopulationestimates).

[15] Quan H, Li B, Couris CM, Fushimi K, Graham P, Hider P, et al. Updating and validating the Charlson comorbidity index and score for risk adjustment in hospital discharge abstracts using data from 6 countries. Am J Epidemiol 2011; 173(6): 676–82. 21330339 10.1093/aje/kwq433

[16] NHS data dictionary. NHS Data Model and Dictionary: Organ System Supported. NHS Digital, 2021 (https://datadictionary.nhs.uk/attributes/organ_system_supported.html).

[17] AbuRuz ME, Momani A, Shajrawi A. The association between depressive symptoms and length of hospital stay following coronary artery bypass graft is moderated by perceived control. Risk Manag Healthc Policy 2021; 14: 1499–507. 33883956 10.2147/RMHP.S306162 PMC8053611

[18] Sugawara N, Metoki N, Hagii J, Saito S, Shiroto H, Tomita T, et al. Effect of depressive symptoms on the length of hospital stay among patients hospitalized for acute stroke in Japan. Neuropsychiatr Dis Treat 2015; 11: 2551–6. 26491334 10.2147/NDT.S91303 PMC4599635

[19] Siddiqui N, Dwyer M, Stankovich J, Peterson G, Greenfield D, Si L, et al. Hospital length of stay variation and comorbidity of mental illness: a retrospective study of five common chronic medical conditions. BMC Health Serv Res 2018; 18(1): 498. 29945622 10.1186/s12913-018-3316-2 PMC6020383

[20] Adams LY, Koop P, Quan H, Norris C. A population-based comparison of the use of acute healthcare services by older adults with and without mental illness diagnoses. J Psychiatr Ment Health Nurs 2015; 22(1): 39–46. 25430792 10.1111/jpm.12169

[21] Germack HD, Noor EAM, Wang X, Hanrahan N. Association of comorbid serious mental illness diagnosis with 30-day medical and surgical readmissions. JAMA Psychiatry 2019; 76(1): 96–8. 30476934 10.1001/jamapsychiatry.2018.3091 PMC6583450

[22] Baker JM, Grant RW, Gopalan A. A systematic review of care management interventions targeting multimorbidity and high care utilization. BMC Health Serv Res 2018; 18(1): 65. 29382327 10.1186/s12913-018-2881-8 PMC5791200

[23] Anderson KK, Fuhrer R, Malla AK. ‘There are too many steps before you get to where you need to be’: help-seeking by patients with first-episode psychosis. J Ment Health 2013; 22(4): 384–95. 22958140 10.3109/09638237.2012.705922

[24] Butler J, de Cassan S, Turner P, Lennox B, Hayward G, Glogowska M. Attitudes to physical healthcare in severe mental illness; a patient and mental health clinician qualitative interview study. BMC Fam Pract 2020; 21(1): 243. 33243139 10.1186/s12875-020-01316-5 PMC7693502

[25] Lomholt LH, Andersen DV, Sejrsgaard-Jacobsen C, Ozdemir CM, Graff C, Schjerning O, et al. Mortality rate trends in patients diagnosed with schizophrenia or bipolar disorder: a nationwide study with 20 years of follow-up. Int J Bipolar Disord 2019; 7(1): 6. 30820700 10.1186/s40345-018-0140-x PMC6395457

[26] Correll CU, Detraux J, De Lepeleire J, De Hert M. Effects of antipsychotics, antidepressants and mood stabilizers on risk for physical diseases in people with schizophrenia, depression and bipolar disorder. World Psychiatry 2015; 14(2): 119–36. 26043321 10.1002/wps.20204 PMC4471960

[27] Correll CU, Rubio JM, Kane JM. What is the risk-benefit ratio of long-term antipsychotic treatment in people with schizophrenia? World Psychiatry 2018; 17(2): 149–60. 29856543 10.1002/wps.20516 PMC5980517

[28] Novick D, Montgomery W, Treuer T, Aguado J, Kraemer S, Haro JM. Relationship of insight with medication adherence and the impact on outcomes in patients with schizophrenia and bipolar disorder: results from a 1-year European outpatient observational study. BMC Psychiatry 2015; 15: 189. 26239486 10.1186/s12888-015-0560-4 PMC4524170

[29] Osborn DP, Nazareth I, King MB. Physical activity, dietary habits and coronary heart disease risk factor knowledge amongst people with severe mental illness: a cross sectional comparative study in primary care. Soc Psychiatry Psychiatr Epidemiol 2007; 42(10): 787–93. 17721669 10.1007/s00127-007-0247-3

[30] Boyd A, Van de Velde S, Vilagut G, de Graaf R, O'Neill S, Florescu S, et al. Gender differences in mental disorders and suicidality in Europe: results from a large cross-sectional population-based study. J Affect Disord 2015; 173: 245–54. 25462424 10.1016/j.jad.2014.11.002

[31] Hardy F, Heyl J, Tucker K, Hopper A, Marcha MJ, Briggs TWR, et al. Data consistency in the English Hospital Episodes Statistics database. BMJ Health Care Inform 2022; 29(1): e100633. 10.1136/bmjhci-2022-100633 PMC9621173 36307148

[32] Heyl J, Hardy F, Tucker K, Hopper A, Marcha MJ, Liew A, et al. Data quality and autism: issues and potential impacts. Int J Med Inform 2022; 170: 104938. 36455477 10.1016/j.ijmedinf.2022.104938

[33] McDonald C. Cigarette smoking in patients with schizophrenia. Br J Psychiatry 2000; 176: 596–7. 10974973

[34] Williams JM, Ziedonis DM, Abanyie F, Steinberg ML, Foulds J, Benowitz NL. Increased nicotine and cotinine levels in smokers with schizophrenia and schizoaffective disorder is not a metabolic effect. Schizophr Res 2005; 79(2–3): 323–35. 15961287 10.1016/j.schres.2005.04.016

[35] Peckham E, Brabyn S, Cook L, Tew G, Gilbody S. Smoking cessation in severe mental ill health: what works? An updated systematic review and meta-analysis. BMC Psychiatry 2017; 17(1): 252. 28705244 10.1186/s12888-017-1419-7 PMC5513129

[36] Gilbody S, Peckham E, Bailey D, Arundel C, Heron P, Crosland S, et al. Smoking cessation for people with severe mental illness (SCIMITAR+): a pragmatic randomised controlled trial. Lancet Psychiatry 2019; 6(5): 379–90. 30975539 10.1016/S2215-0366(19)30047-1 PMC6546931

