# Supplementary material for: Comparison of outcomes for patients with and without a serious mental illness presenting to hospital for chronic obstruction pulmonary disease: retrospective observational study using administrative data
Source: BJPsych Open. 2023 Jul 17;9(4):e128. doi: 10.1192/bjo.2023.522 (PMC10375884; doi:10.1192/bjo.2023.522)
Supplement: Supplementary file 1 [file S2056472423005227sup001.docx]

**Supplementary Table S1: Definitions for International Statistical Classification of Disease and Related Health Problems 10th edition (ICD-10) codes**

| **ICD-10 code** | **Description** |
| --- | --- |
| ***Psychosis spectrum disorders*** | |
| F20 | Schizophrenia |
| F200 | Paranoid schizophrenia |
| F201 | Hebephrenic schizophrenia |
| F202 | Catatonic schizophrenia |
| F203 | Undifferentiated schizophrenia |
| F204 | Postschizophrenic depression |
| F205 | Residual schizophrenia |
| F206 | Simple schizophrenia |
| F208 | Other schizophrenia |
| F209 | Schizophrenia, unspecified |
| F21 | Schizotypal disorder |
| F22 | Persistent delusional disorders |
| F220 | Delusional disorder |
| F228 | Other persistent delusional disorders |
| F229 | Persistent delusional disorder, unspecified |
| F23 | Acute and transient psychotic disorders |
| F230 | Acute polymorphic psychotic disorder without symptoms of schizophrenia |
| F231 | Acute polymorphic psychotic disorder with symptoms of schizophrenia |
| F232 | Acute schizophrenia-like psychotic disorder |
| F233 | Other acute predominantly delusional psychotic disorders |
| F238 | Other acute and transient psychotic disorders |
| F239 | Acute and transient psychotic disorder, unspecified |
| F24 | Induced delusional disorder |
| F25 | Schizoaffective disorders |
| F250 | Schizoaffective disorder, manic type |
| F251 | Schizoaffective disorder, depressive type |
| F252 | Schizoaffective disorder, mixed type |
| F258 | Other schizoaffective disorders |
| F259 | Schizoaffective disorder, unspecified |
| F28 | Other nonorganic psychotic disorders |
| F29 | Unspecified nonorganic psychosis |
| ***Bipolar disorder*** | |
| F30 | Manic episode |
| F300 | Hypomania |
| F301 | Mania without psychotic symptoms |
| F302 | Mania with psychotic symptoms |
| F308 | Other manic episodes |
| F309 | Manic episode, unspecified |
| F31 | Bipolar affective disorder |
| F310 | Bipolar affective disorder, current episode hypomanic |
| F311 | Bipolar affective disorder, current episode manic without psychotic symptoms |
| F312 | Bipolar affective disorder, current episode manic with psychotic symptoms |
| F313 | Bipolar affective disorder, current episode mild or moderate depression |
| F314 | Bipolar affective disorder, current episode severe depression without psychotic symptoms |
| F315 | Bipolar affective disorder, current episode severe depression with psychotic symptoms |
| F316 | Bipolar affective disorder, current episode mixed |
| F317 | Bipolar affective disorder, currently in remission |
| F318 | Other bipolar affective disorders |
| F319 | Bipolar affective disorder, unspecified |
| ***Chronic obstructive pulmonary disease*** | |
| J40 | Bronchitis, not specified as acute or chronic |
| J41 | Simple and mucopurulent chronic bronchitis |
| J410 | Simple chronic bronchitis |
| J411 | Mucopurulent chronic bronchitis |
| J418 | Mixed simple and mucopurulent chronic bronchitis |
| J42 | Unspecified chronic bronchitis |
| J43 | Emphysema |
| J430 | MacLeod's syndrome |
| J431 | Panlobular emphysema |
| J432 | Centrilobular emphysema |
| J438 | Other emphysema |
| J439 | Emphysema, unspecified |
| J44 | Other chronic obstructive pulmonary disease |
| J440 | Chronic obstructive pulmonary disease with acute lower respiratory infection |
| J441 | Chronic obstructive pulmonary disease with acute exacerbation, unspecified |
| J448 | Other specified chronic obstructive pulmonary disease |
| J449 | Chronic obstructive pulmonary disease, unspecified |
